# Asymptomatic hemilateral pneumothorax and pneumomediastinum following surgical tracheostomy in a patient with hyponatremia and zolpidem withdrawal delirium

**DOI:** 10.1186/s40981-018-0166-1

**Published:** 2018-04-03

**Authors:** Yoshihiro Takasugi, Risa Aoki, Shota Tsukimoto

**Affiliations:** Department of Anesthesiology, Kindai University Faculty of Medicine, 377-2 Ohno-higashi, Osaka-sayama, Osaka 589-8511 Japan

**Keywords:** Tracheostomy, Pneumothorax, Pneumomediastinum, Delirium, Hyponatremia

## To the editor

Pneumothorax following tracheostomy is a rare complication, with a reported incidence of approximately 1% following both open surgical and percutaneous tracheostomy [1, 2], but potentially life-threatening complication. Post-tracheostomy pneumothorax can usually be diagnosed by clinical signs and symptoms even before a postoperative chest film is obtained. We report a case of asymptomatic pneumothorax and pneumomediastinum following tracheostomy in an adult patient with delirium, but was diagnosed by radiographic examinations of the next day.

## Case presentation

A 59-year-old man was admitted to our acute care unit due to persistent intraoral bleeding following extraction of a wisdom tooth. The patient habitually took 20 mg/day of zolpidem, imidazopyridine class of γ-aminobutyric acid A (GABA_A_) receptor agonist, often taking more than 50 mg/day. Laboratory tests indicated hyponatremia (Na 123 mEq/L, serum osmolality 256 mOsm/L) secondary to the syndrome of inappropriate secretion of antidiuretic hormone (SIADH) (ADH 45.3 pg/mL). At the time of admission, he mumbled in delirium and developed generalized tonic seizures twice.

At 11 h after his admission, he vomited approximately 200 mL of clot and blood, and SpO_2_ decreased to 80%; since aspiration due to intraoral bleeding was suspected, emergency hemostasis and tracheostomy for postoperative respiratory management were performed. Two hours later, SpO_2_ was 100% with 5 L/min oxygen through the tracheostomy mask. We continuously infused 3% hypertonic saline and administered fosphenytoin and diazepam for treatment of hyponatremia and generalized tonic seizures, respectively. With this treatment, hyponatremia improved and generalized tonic seizures were relieved, while delirium and confusion lasted.

**Fig. 1:**
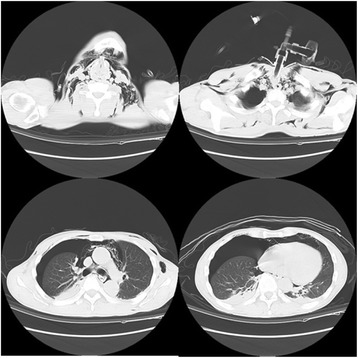
CT images of the neck and thorax on the day following tracheostomy. CT images revealed emphysema of the chest, neck and mediastinum, and pneumothorax of the right lung

On the second hospital day, he complained of pain in his head and neck together with mild dyspnea (SpO_2_ 95–96% with 2 L/min oxygen). Hemilateral pneumothorax, pneumomediastinum, and subcutaneous emphysema were diagnosed by chest X-ray and CT images of the thorax and neck, for which a chest tube was inserted (Fig. 1). On the seventh hospital day, CT images indicated disappearance of the pneumothorax and remission of subcutaneous emphysema and pneumomediastinum, and the chest tube was removed. On the ninth hospital day, his consciousness and communication normalized and he was discharged on the twelfth hospital day.

## Discussion

The diagnosis of post-tracheostomy pneumothorax is usually based on clinical signs and symptoms, such as sudden and acute chest pain, dyspnea, tachycardia, decreased breath sounds and oxygen desaturation, and routine use of post-tracheostomy chest radiography has currently been reported as unnecessary in adults [3–5]. In this case, when tension pneumothorax and pneumomediastinum were diagnosed by chest X-ray and CT examinations, the patient did not have any of the usual features of both pneumothorax and pneumomediastinum; he only complained of pain in his head and neck, and his SpO_2_ was 95–96% while breathing 2 L/min oxygen. Symptoms of hyponatremia and zolpidem withdrawal include delirium, confusion, disorientation, and seizures [6, 7]. Patients with delirium exhibit symptoms of disturbances in consciousness, orientation, memory, perception, and behavior: tactile hallucinations in patients with delirium may involve paresthesia and pain [8]. We assumed that our patient’s sensory disturbance associated with the delirium may have resulted in the absence of clinical signs of tension pneumothorax.

## Conclusion

The current case suggests that postoperative radiographic examinations may have a role to play in the early diagnosis of pneumothorax and pneumomediastinum in patients with delirium.

## Abbreviations

ADH: Antidiuretic hormone
CT: Computed tomography
GABA_A_: γ-Aminobutyric acid A
SIADH: Syndrome of inappropriate secretion of antidiuretic hormone
SpO_2_: Percutaneous oxygen saturation
